# Editorial: Psychological impact and quality of life in rheumatic and musculoskeletal diseases

**DOI:** 10.3389/fmed.2023.1180240

**Published:** 2023-03-22

**Authors:** Rosaria Talarico, Anca D. Askanase, Maurizio Cutolo

**Affiliations:** ^1^Rheumatology Unit, Azienda Ospedaliero Universitaria Pisana, Pisa, Italy; ^2^Department of Rheumatology, Columbia University Medical Center, New York, NY, United States; ^3^Laboratory of Experimental Rheumatology and Academic Division of Clinical Rheumatology, Department of Internal Medicine, IRCCS San Martino Polyclinic Hospital, University of Genova, Genoa, Italy

**Keywords:** psychological impact, quality of life, rheumatic diseases, psychological distress, systemic autoimmune rheumatic disease

Rheumatic diseases are generally characterized by a chronic course that both affects patients' physical, mental, and social function and significantly impacts their caregivers and family members, resulting in low health-related quality of life (QoL) as well as psychological problems (1, 2). For this reason, over the last decade, health related QoL has become an evolving field of research; and data from the literature increasingly highlight the relationship between disease activity/damage and the ability to work and psychological wellbeing.

Alongside these considerations, it is important to point out that exploring QoL in chronic rheumatic diseases can be extremely complex, disease-specific tools are not always available, and are not able to detect the patients' perspective as it relates to defined disease area (3, 4).

The articles included in the current Research Topic bring further insights into the burden of psychological impact and QoL in patients affected by chronic rheumatic diseases.

It is indeed crucial to address the needs of patients at 360 degrees of their lives, in order to ensure that all aspects of the patients' lives are taken into account in the management of their diseases Unger et al. sought to identify the range of perspectives of patients with primary Sjoegren's syndrome (pSS) from five European countries on function and health to uncover commonalities and better understand their experiences. Using focus groups, the authors combined key concepts from each country and mapped them to the International Classification of Functioning, Disability and Health (ICF). Findings from a large number of pSS patients who participated in focus groups indicate significant limitations in daily living due to a mismatch between the person's abilities, environmental demands, and activity requirements; these perspectives should therefore be considered when assessing the quality of life of pSS patients in clinical care.

Anxiety and depression symptoms are common in patients with systemic lupus erythematosus (SLE), including outpatients with mild to moderate disease. Elefante et al. examined the extent to which such symptoms have a significant negative impact on QoL and perceptions of disease activity. The Hospital Anxiety and Depression Scale (HADS) was used to self-assess anxiety and depression. Data from a cohort of SLE patients showed that fibromyalgia and older age were independently associated with anxiety and depression. Active skin involvement was also significantly associated with depression. Higher scores on the HADS questionnaire (higher anxiety and depression scores) correlated significantly with patients' perceived higher disease activity and poorer quality of life.

Stress is common in SLE and is associated with depression, fatigue, and disease flares; Jolly and Katzhttps explored predictors of stress in SLE patients. The authors observed that high stress level was associated with younger age, worse QoL, and presence of comorbidities; moreover, the presence of stress at baseline seems to be predictive of persistent stress during follow-up. Considering that stress could be modifiable, identifying patients at high risk could allow clinicians, and health care professionals to develop a multidisciplinary management strategy and appropriately direct resources.

Lindblom et al. investigated the role of health state assessment in predicting the accrual of organ damage. Using data from the open-label extension of the belimumab trials BLISS-52 and BLISS-76, the authors probed whether EQ-5D full health state (FHS) after therapy was associated with a reduced risk of damage accrual in SLE patients. Moreover, the association between experiencing “no problems” in each one of the five dimensions of EQ-5D and the risk to accrue damage was assessed. EQ-5D-3L FHS and “no problems” with mobility after therapeutic intervention indicated a lower risk of suffering subsequent organ damage, especially musculoskeletal. This suggests that optimization of these health-related QoL aspects, in addition to clinical and laboratory parameters, is a clinically relevant treatment goal in patients with SLE.

Assessing QoL in daily care can be challenging, especially considering the recent difficulties experienced by health care systems during the COVID-19 pandemic (5). In this scenario, telemedicine became a useful alternative to traditional face-to-face evaluations, allowing clinicians and health care professionals to manage patients, including their QoL. Tang et al. described patient and provider experiences with telemedicine and its impact on care. Consistent numbers of physician visits and admissions and similar quality of life between pandemic and pre-pandemic levels suggest effective treatment of rheumatic diseases with combined telemedicineand in-person care.

Finally, Trieste et al. conducted a state-of-the-art study aimed at better understanding QoL metrics used in clinical and economic research to assess individual perspectives on living with rare and complex connective tissue diseases. Anxiety, depression, body image satisfaction, daily activity, fatigue, illness perception, pain, personality, quality of life, resilience, relationship satisfaction, self-management, sexual quality of life, sleep quality, social support, stress, uncertainty, and work productivity are the most commonly used dimensions in the literature. However, even in diseases characterized by relatively high prevalence and incidence, such as SLE, pSS, and systemic sclerosis, analysis of patient resilience, satisfaction with relationship quality, personality, and stress seems to be underrepresented.

We acknowledge that in complex conditions, like SLE (6) or systemic vasculitis, psychiatric/psychological disturbances may additionally arise from direct disease related brain involvement and need to be carefully investigated, requiring multidisciplinary team interventions.

In conclusion, the psychological impacts and the effects on quality of life in patients with chronic rheumatic diseases are complex and difficult to explore. The data included here suggest that more work needs to be done to improve the assessment and evaluation of the impact and QoL issues in order to better educate patients and providers (7). We need to continue our efforts to longitudinally assess the psychological impact and QoL in patients with musculoskeletal and rheumatic diseases. Including patients in the design and evaluation of studies aimed at defining this impact will advance our assessment tools (including specific questionnaires aimed at exploring the unmet needs of people affected by rheumatic diseases). Ultimately, this will improve the lives of people living with chronic rheumatic diseases.

## Author contributions

All authors listed have made a substantial, direct, and intellectual contribution to the work and approved it for publication.
